# Emerging Roles of Receptor-like Protein Kinases in Plant Response to Abiotic Stresses

**DOI:** 10.3390/ijms241914762

**Published:** 2023-09-29

**Authors:** Akanksha Gandhi, Ralf Oelmüller

**Affiliations:** Matthias Schleiden Institute of Genetics, Bioinformatics and Molecular Botany, Department of Plant Physiology, Friedrich-Schiller-University, 07743 Jena, Germany; agandhi@ice.mpg.de

**Keywords:** receptor-like kinases, receptor-like cytoplasmic kinases, abiotic stresses, reactive oxygen species, signaling

## Abstract

The productivity of plants is hindered by unfavorable conditions. To perceive stress signals and to transduce these signals to intracellular responses, plants rely on membrane-bound receptor-like kinases (RLKs). These play a pivotal role in signaling events governing growth, reproduction, hormone perception, and defense responses against biotic stresses; however, their involvement in abiotic stress responses is poorly documented. Plant RLKs harbor an N-terminal extracellular domain, a transmembrane domain, and a C-terminal intracellular kinase domain. The ectodomains of these RLKs are quite diverse, aiding their responses to various stimuli. We summarize here the sub-classes of RLKs based on their domain structure and discuss the available information on their specific role in abiotic stress adaptation. Furthermore, the current state of knowledge on RLKs and their significance in abiotic stress responses is highlighted in this review, shedding light on their role in influencing plant–environment interactions and opening up possibilities for novel approaches to engineer stress-tolerant crop varieties.

## 1. Introduction

Plants are continuously challenged by biotic and abiotic stresses. Being sessile, they cannot escape from these threats, and they must be able to perceive, process and respond in a timely and efficient manner. Abiotic stresses, such as low or high temperature, drought, high salinity, or the presence of heavy metals, affect their physiology, growth and development, thus hampering agricultural productivity. It is estimated that abiotic stresses lead to more than 50 percent of yield losses in most plant species [1,2]. Recognition of the stress signal initiates changes at the cellular and molecular levels leading to transcriptional reprogramming which is instrumental for their growth under diverse conditions. Thus, it becomes crucial to understand how different environmental stimuli are perceived and translated into signaling events [3,4]. Overall, stress response is orchestrated by a plethora of signaling molecules, such as ion channels, transcription factors, protein kinases and phosphatases [3,5], and secondary messengers such as calcium, cyclic nucleotides or reactive oxygen species (ROS) [6,7].

**Figure 1:**
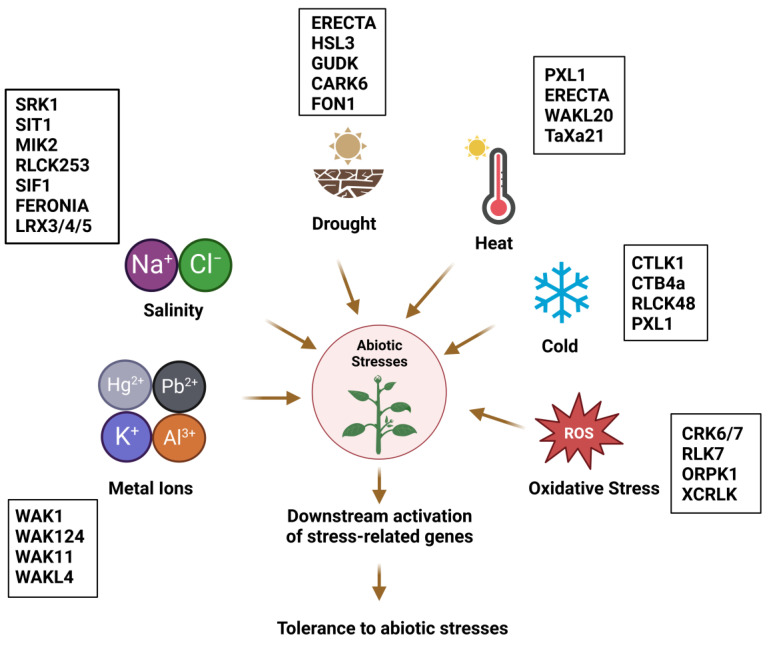
Overview of the role of plant RLKs in diverse abiotic stress responses with representation of some examples. For details and abbreviations, cf. text.

It has been well established that protein kinases such as mitogen-activated protein kinases (MAPKs), receptor-like kinases (RLKs), sucrose nonfermenting 1-related protein kinases, and calcium-dependent protein kinases, play a vital role in modulating plant growth and development during abiotic stress [8]. Eukaryotic protein kinases (ePKs) can phosphorylate serine, threonine or tyrosine residues of their substrates, thus changing their activities. [9,10]. ePKs are a highly complex superfamily consisting of 1.5–2.5% of all eukaryotic genes [11]. Kinome is the term used to describe the complete set of protein kinases encoded in a genome [12]. In plants, RLKs form one of the largest gene families that share structural similarity with animal receptor tyrosine kinases [13], and their closest homologs are *Drosophila melanogaster* Pelle [14] and mammalian interleukin receptor-associated kinases [15,16]. A typical RLK harbors a highly variable extracellular domain for ligand binding, a transmembrane domain and a cytoplasmic kinase domain for signal propagation [17,18]. RLKs play a pivotal role in sensing developmental cues, mediating plant growth, reproduction, stomatal patterning, pollen tube guidance, symbiosis, hormone signal transduction [19,20,21], and adaptation to biotic and abiotic stresses [22,23,24] (Figure 1). For instance, the leucine-rich repeat receptor-like protein kinase (LRR-RLK) brassinosteroid insensitive 1 (BRI1) regulates root elongation, seed germination, stomatal function, pathogen attack and senescence and is also involved in various abiotic stresses, such as abnormal temperatures, drought and high osmotic pressure [19,25,26,27,28]. In *Arabidopsis thaliana*, overexpression of lectin-like protein kinase 1 (AtLPK1) results in enhanced resistance to infection by the necrotrophic fungus *Botrytis cinerea* as well as improved seed germination and cotyledon greening under high salt stress, indicating that it is vital for both biotic and abiotic stress responses [29].

Genome analyses have shown the presence of 610 RLKs in *A. thaliana* and over 1131 in rice [17]. Interestingly, an RLK subfamily that possesses only the cytoplasmic kinase domain has been designated as a receptor-like cytoplasmic kinase (RLCK) family. There are 200 RLCKs in Arabidopsis and 379 in rice [17,30]. RLCKs are attached to the plasma membrane through N-terminal putative myristoylation and/or palmitoylation motifs [31,32]. Studies have also reported their association with RLKs and their role as regulatory elements to relay intracellular signals via transphosphorylation [33,34,35]. Recent advances suggest their role in hormone signaling pathways, plant immune responses, growth and development, embryonic patterning and organ abscission but their biological function in abiotic stress responses has not been well investigated [36,37,38]. Boisson-Dernier et al. [39] have reported that MARIS (MRI), a member of the RLCK-VIII subfamily, functions downstream of ANXUR1 and -2 which are RLKs of the *Catharanthus roseus* RLK1-like (*Cr*RLK1L) subfamily. Activation of the ROS-generating NADPH oxidases regulate root hair growth and cell wall integrity of pollen tubes in *A. thaliana.* Botrytis-induced kinase 1 (BIK1), a RCLK VII member, forms a complex with both flagellin sensing 2 (FLS2) and brassinosteroid insensitive1-associated kinase 1 (BAK1) to activate microbe-associated molecular patterns (MAMP) triggered immunity [18,31,40,41]. However, there is also evidence that the FLS2 is involved in abiotic stress tolerance [42].

Much research has been conducted that provides fundamental insights into mechanisms by which different RLKs activate defenses against microbes [43,44,45,46]. However, only a handful of studies have shed light on the role of RLKs in abiotic stress responses and the potential mechanisms underlying RLK-mediated abiotic stress tolerance [47,48]. A deeper understanding of kinase signaling cascades in responses to fluctuating environmental conditions such as drought, heat, cold, or salt is paramount to engineer stress-tolerant crops [49]. This review provides insights into the classification of RLKs and the biological functions of RLKs and RLCKs in coordinating the responses of different plant species against abiotic stresses such as salinity, drought and oxidative stress. Furthermore, this review also highlights arising avenues of future research to unravel novel targets of plant stress responses.

## 2. Classification of Arabidopsis RLKs

On the basis of signature motifs in their extracellular domains, RLKs are categorized into 14 classes: [50]: leucine-rich repeat (LRR), lectin (C-Lectin and L-Lectin), wall-associated kinase (WAK), extension-like, proline-rich extension-like (PERK), CrRLK, self-incompatibility domain (S-domain), CRINKLY4-like (CR-like), the domain of unknown function 26 (DUF26), lysin motif (LysM), thaumatin, leaf rust kinase-like (LRK), receptor-like kinase in flowers (RKF), and chitinase (glycoside hydrolase)-type domain proteins [51]. Such a diversity makes the RLK family one of the most versatile gene families and enables them to react to a variety of external stimuli by binding to proteins, polysaccharides, lipids and other ligands [17,51,52].

### 2.1. Leucine-Rich Repeat-Receptor-like Kinases (LRR-RLKs)

LRR-RLKs form the largest RLK subfamily in *A. thaliana,* consisting of 239 genes and 15 subfamilies based on the amino acid relationships between their kinase domains [13,53]. The LRR is a tandem repeat of 24 amino acids with conserved leucines [54,55]. Genetic and biochemical experiments have shown that LRR-RLKs can recognize various ligands such as small molecules [56,57,58], peptides and entire proteins [59,60]. These receptors often form heterodimers with other LRR units that act as co-receptors, such as BAK1 or suppressor of BAK1-interacting receptor-like kinase1 (BIR1)-1 (SOBIR) for the activation of signaling cascades [61,62,63,64,65]. LRR-RLKs in Arabidopsis are involved in cell division, proliferation, differentiation, stem cell balance, pathogen resistance, hormone perception and stress adaptation [20,66,67] (Table 1). Considering abiotic stress, Pitorre et al. [68] uncovered a role of RLK7 in oxidative stress tolerance in *A. thaliana*. By generating knockout and overexpressing lines, *Oryza sativa* stress-induced protein kinase gene 1 (OsSIK1) was reported to play a positive role in salinity and drought stress by the upregulation of antioxidative enzyme activities. The leaves of OsSIK1-overexpressing plants accumulated less H_2_O_2_ than those of mutants and control plants [69]. Yang et al. [70] identified a novel LRR-RLK from *Glycine soja* (GsLRPK) that is involved in cold signaling. Overexpression of GsLRPK in yeast and Arabidopsis improved cold tolerance by stimulating the expression of cold-inducible marker genes such as *Kinase 1* (*KIN1*) and *Cold regulated 15b* (*COR15b*).

### 2.2. Lectin Domain-Containing Receptor-like Kinases (LecRLKs)

Lectins are widespread proteins that contain at least one noncatalytic domain with the capability to bind reversibly to a specific mono- or oligosaccharide [71]. A noxious protein called ricin was found in the seeds of the castor bean (*Ricinus communis* L.) in 1888 that was later identified as the first lectin [72]. The LecRLK family consists of 75 members in Arabidopsis and 173 members in rice [73]. Few lectins, such as the calnexins, calreticulins and malectins that are responsible for protein folding in the endoplasmic reticulum, are prevalent in plants, fungi, and animals [74,75,76,77,78]. LecRLKs are classified into several groups based on their structural features and sequence similarities. L-type LecRLKs harbor a legume–lectin protein-like extracellular domain [79]. The extracellular domain of B-type lectins resembles the bulb–lectin proteins and were renamed as GNA-related (*Galanthus nivalis* agglutinin-related) or G lectins. Earlier, they were called S-domain RLKs as they contain an S-locus that participates in a self-incompatibility reaction [80]. There is the presence of an epidermal growth factor (EGF) domain and/or a PAN motif which is potentially crucial for protein–protein and protein–carbohydrate interactions [81,82,83]. While C-type (calcium-dependent) lectin motifs are present in mammalian proteins that regulate innate immune responses [84,85], plant LecRKs function in pollen development, pathogen resistance [86,87,88], wounding [89,90], regulation of stomatal immunity [91], herbivory [92,93] and tolerance to abiotic stresses [94]. An Arabidopsis *LecRK-b2* which is expressed during seed germination positively regulates abscisic acid (ABA), salt and osmotic stress responses [95]. *Pohlia nutans* lectin-like protein kinase 1 (PnLecRLK1) that localizes at the plasma membrane has been shown to enhance chilling stress tolerance and ABA sensitivity which allows Antarctic mosses to survive under extreme conditions. Moreover, its expression was induced by various abiotic stresses, including cold, salt, drought, ABA, and methyl jasmonic acid (MeJA) treatments. PnLecRLK1-overexpressing lines showed elevated transcription levels of genes in the C-repeat binding factor (CBF) signaling pathway, such as AtCBF1, AtCBF2, AtCBF3 and AtCOLD RESPONSIVE47 (AtCOR47) [96].

### 2.3. Wall Associated Kinases (WAKs) 

WAKs physically connect the extracellular matrix to the plasma membrane and allow communication between the two subcellular compartments [97,98]. These receptors can perceive the pectin breakdown products, oligogalacturonides [99]. Five WAK isoforms (WAK1–WAK5), clustered in 30 kilobases, share 40 to 64% similarity in their extracellular amino terminal and 86% in their cytoplasmic kinase domains [100]. WAKs encompass epidermal growth factor (EGF)-like repeats at the amino terminal side adjacent to the transmembrane domains [101]. In Arabidopsis, 26 *WAK-like* (*WAKL*) genes have been discovered due to their sequence similarity to *WAKs* [102,103]. Intriguingly, rice has 125 WAKs [104], barley has 91 WAKs [105], maize has more than 100 WAKs [106] and *Brachypodium distachyon* has 115 WAKs [107]. WAK1, −2, −3, and −5 are expressed in green organs; WAK1 and −2 are expressed in flowers and siliques; and WAK2 is expressed in roots, while WAK4 is only expressed in siliques [108]. Besides their involvement in cell expansion, development, wounding and pathogen invasion, their expression is also stimulated by heavy metals, and ozone [98,100,109,110,111]. Bot et al. [112] investigated the role of AtWAKL10 in response to nitric oxide (NO) and its involvement in plant defense against pathogens and abiotic stresses. Using transcriptome analysis, the mRNA levels of WAKL10 changed in response to the NO donor S-nitroso-L-cysteine (CysNO). Both oxidative and nitrosative stresses had distinct effects on the growth of *atwakl10* plants; CysNO treatment resulted in a greater growth rate but S-nitrosoglutathione and methyl-viologen treatment ceased its growth. AtWAKL10 positively regulates salt stress but has adverse effects under drought conditions. Analysis of the promoter region confirmed the presence of *cis*-regulatory elements that are crucial for abiotic stress tolerance. These results add to our understanding of the mechanisms by which plants can ameliorate stress based on the intricate interactions between NO signaling and AtWAKL10. Furthermore, AtWAKL10 could be an interesting protein to be investigated for the crosstalk between NO-induced responses to biotic and abiotic stress.

### 2.4. Lysin Motif Receptor-like Kinase Family (LysM-RLKs)

LysM, about 44–65 amino acids long, was first discovered in the antimicrobial protein, lysozyme, of *Bacillus* phage ϕ29 [113]. X-ray crystallography and homology modelling uncovered the three-dimensional βααβ structure of LysM that consists of two α-helices stacked on one side of a two-stranded antiparallel β-sheet [114,115,116]. Their extracellular region is composed of three LysM modules separated by cysteine pair motifs (CxC) which are required for disulfide bridge formation and stabilization of the protein structure [117,118]. This kinase family is a second major class of plant pattern recognition receptors that can recognize proteinaceous microbial patterns such as short and long chitooligomers, lipochitooligomers, and peptidoglycans, mainly *N*-acetyl glucosamine [119,120,121,122,123]. These RLKs can detect both symbiotic and pathogenic microorganisms. It has been reported that the *A. thaliana* genome contains five LysM-RLKs. LYK1/CERK1, a classical LysM member, is implicated in chitin recognition, which is a component of the fungal cell wall. Additionally, LYK4 and LYK5 are also necessary for the formation of heterodimers to activate chitin-mediated immune responses [124,125,126]. In the past few years, substantial progress has been made in elucidating the signaling pathways and gene networks of LysM-RLKs in plant immune activation and symbiosis, but we are still far away from understanding their role in abiotic stresses.

Espinoza et al. [127] have unraveled the molecular mechanism that contributes to cross-tolerance between chitin and salt. Interestingly, *CERK1* expression was upregulated under salt stress and the *cerk1* mutant was more prone to salt stress but was not affected by osmotic stress. Transcriptome studies have shown similar expression profiles of chitin and salt treatments. It would be interesting to investigate the link between chitin-mediated plant defense responses and salt stress responses. Moreover, *cerk1* plants have shown an aberrant rise in the levels of cytosolic calcium ([Ca^2+^]_cyt_) after salt application. Bimolecular fluorescence complementation and co-immunoprecipitation experiments have revealed the interaction between CERK1 and ANNEXIN1, a Ca^2+^-permeable channel that mediates the salt-elicited [Ca^2+^]_cyt_ signal [128]. The above evidence helps us to comprehend the crosstalk between biotic and abiotic signaling pathways. Furthermore, the question as to which Ca^2+^-channels are involved in biotic and abiotic signaling and whether there is a crosstalk is still enigmatic.

### 2.5. Cysteine-Rich Repeat Domain-Containing Receptor-like Kinases (CRKs)

Most CRKs have two domain of unknown function 26 (DUF26) motifs in their extracellular region with three conserved cysteine (C) residues in a C-X8-C-X2-C configuration [129,130,131]. These conserved C residues are postulated to be involved in the formation of disulfide bridges to stabilize the three-dimensional structure of these kinases or in ROS redox regulation [130,131,132]. The DUF26 domain has an antifungal property which is why it is also called a stress–antifungal domain (PF01657) [133,134,135] or GINKBILOBIN2 (GNK2), and its role in salt stress response has also been reported [136]. Multi-omics and molecular genetic analyses have led to the identification of 44 CRKs in *A. thaliana* and 1074 CRKs in 14 other crops. However, the role of only 63 members has been characterized and the function of the other members is still obscure [137].

Functional characterization of CRKs has unveiled their involvement in development [138,139,140], defense [141,142,143,144,145,146], cell death [147,148,149], acclimation to various abiotic stresses such as salt [150,151,152], osmotic stress [153], UV light, oxidative [129], heat [154], cold stress [155] and drought [131,156,157]. In addition, existing evidence shows the upregulation of CRKs by O_3_ [131,158], salicylic acid (CRK4, CRK5, CRK6, CRK10, CRK11, CRK19, CRK20, CRK45), pathogens and their microbial patterns [147,148,159,160]. Bioinformatic studies have revealed the presence of W-Box elements (TTGAC) which are the binding sites for WRKY transcription factors in the promoters of *AtCRKs,* implying that WRKYs play a role in the regulation of these kinases [131,139,148,161,162,163].

The presence of conserved C residues points towards the role of CRKs in ROS signaling. Bourdais et al. [139] analyzed the effect of chloroplast ROS inducers, methyl viologen (paraquat) and 3-(3,4-dichlorophenyl)-1,1-dimethylurea (DCMU), on different *crk* mutants, and higher photoinhibition was observed in *crk2*, *crk5*, *crk8*, *crk17*, *crk20*, *crk40*, and *crk45* mutants compared with the wild-type. Furthermore, *crk2* and *crk45* mutants also exhibited higher electrolyte leakage upon exposure to light stress. Another study demonstrated the role of CRK2 in improving germination, root length, and callose deposition under high salt stress, thereby suggesting that it is necessary for salt tolerance [150].

### 2.6. CRINKLY4 (CR4) Family of Receptor-like Kinases

These kinases were first described in maize where the *CR4* gene was isolated by mutator–transposon tagging. *cr4* mutants were shorter, had crinkly leaves, and had defects in the differentiation of the leaf epidermis [164]. In addition, the epidermal cells of these mutants were aberrant in shape, cell wall thickness, cuticle formation, vesicle trafficking and showed tumor-like outgrowths [164,165]. The characteristic feature of this family is the presence of 7 crinkly repeats of 39 amino acids in the extracellular region and a domain homologous to tumor necrosis factor receptor (TNFR) cysteine-rich region [164,166]. Arabidopsis CR4 (ACR4), an ortholog of CR4, contains all of the features of maize CR4 and is strongly expressed in the protodermal cells of the embryo, in the L1 layer of the shoot apical meristem, in the epidermis of leaf primordia, in the small daughter cells after the first asymmetric pericycle cell division and in the root stem cell niche [167,168,169,170]. Localization studies in *Nicotiana benthamiana* leaf epidermal cells have revealed the accumulation of ACR4 at the plasmodesmata [171]. Mounting evidence supports the idea of a role for ACR4 in regulating asymmetric cell division in columella stem cells, embryonic development, and lateral root formation [168,169,170,171,172,173,174,175]. Furthermore, ACR4 and an LRR-RLK, CLAVATA1, can form homo- and heterodimers and participate in the maintenance of root meristem in response to the signaling peptide clavata 3/embryo surrounding region 40 (CLE40) [171].

Interestingly, Zereen and Ingram [176] have demonstrated that the *acr4* knockout line is less susceptible to the necrotrophic fungal pathogen *Botrytis cinerea,* and the expression of *ACR4* is significantly downregulated in response to this fungus. It is speculated that this might be due to an enhanced expression of *LIPOXYGENASE2,* which codes for an important enzyme involved in the biosynthesis of jasmonic acid [177]. This finding indicates that it is potentially involved in plant defense responses. While much is known about the role of these kinases in plant growth and development, their role in abiotic stress responses has not been explored. Knowledge of their regulatory networks and downstream signaling components warrants future investigations.

### 2.7. Proline-Rich Extensin like Kinases (PERKs)

The PERK family of kinases consist of an extensin-like extra-cellular domain rich in prolines followed by a typical transmembrane, serine/threonine kinase domain and these were first discovered in *Brassica napus* (*BnPERK1*; [178,179]). This family has fifteen members in Arabidopsis, eight in *Oryza sativa*, two in *Hordeum vulgare*, six in *Solanum tuberosum*, and nineteen in *Brassica rapa* [178,180]. *AtPERK* members have differential expression patterns, most of which are widely expressed while some are expressed in certain tissues. For instance, *AtPERK1* is expressed predominantly in the vascular tissues of cotyledons, developing leaves, and roots; *AtPERK2* is expressed in rosette leaf veins, stems, and pollen [181]; *AtPERK8* and *AtPERK13* in root hairs [179,182,183]; and *AtPERK5*, *AtPERK6*, *AtPERK7*, *AtPERK11*, and *AtPERK12* in pollen [183,184,185].

*AtPERKs* play multifaceted roles in plants, including in the regulation of apical dominance, cell proliferation, and root or pollen tube growth [183,184,185,186]. Additionally, they might also be promising candidates for sensing cell wall integrity, as their extracellular domain resides inside the cell wall, similar to WAKs [178]. Their role in plant–pathogen interactions, abiotic stresses, and hormone signaling has emerged only recently [187,188]. Feng et al. [189] have pinpointed the role of *ZEBRA LEAF 15* (*Z15*), a single copy gene that encodes a PERK, in moderate low-temperature signaling in rice. *Z15* mutation hampered the cell development and chloroplast structure, leading to transversely striped leaves with yellow-green or white-green sectors. Further, the expression of the downstream cold-response genes *OsWRKY71* and *OsMYB4* has also been affected in the *z15* mutant upon exposure to moderate low temperature (18 °C). Recently, *PERK13* was shown to participate in root hair growth under phosphate deficiency. Knocking out and overexpressing *PERK13* extended the root hair elongation period and led to higher ROS generation in root hairs under phosphate-limiting conditions [190].

To date, ligands for these receptor kinases have not been reported, owing to the redundancy of these kinase genes [181,191]. These studies link PERK13-controlled developmental programs to abiotic stress.

### 2.8. Catharanthus Roseus RLK1-like Kinases (CrRLK1Ls)

This family obtained its name after the first identified member, CrRLK1, from *Madagascar periwinkle* (*C. roseus*). Family members have been discovered in different species, including angiosperms (monocots, eudicots), gymnosperms, and early diverging lineages [78,192,193,194]. These receptors consist of two malectin-like domains (MLDs) in their extracellular region that are homologous to the carbohydrate-binding malectin protein from *Xenopus laevis* [21,39,77]. CrRLK1s have diverse functions including responses to biotic and abiotic stresses [47], plant growth [195], morphogenesis [196], reproduction [197], immunity [198], hormone signal transduction [199], RNA metabolism [200] and energy production [201]. Seventeen CrRLK1L members are present in *A. thaliana*, twenty-three in *S. lycopersicum*, twenty in *O. sativa*, and forty in *Gossypium hirsutum* [202,203,204,205].

Eight of the seventeen Arabidopsis CrRLK1Ls have been well investigated to date: FERONIA (FER), ANXUR1 (ANX1), ANXUR2 (ANX2), HERCULES1 (HERK1), HERCULES2 (HERK2), [Ca_2_^+^]_cyt_ -associated protein kinase 1 (CAP1/ERULUS), THESEUS1 (THE1), and CURVY1 (CVY1) [21]. Among these, FER is probably the most extensively characterized CrRLK1L. FER is named after the Etruscan goddess of fertility [206]. It serves as a sensor for salt, osmotic, heat, and cold stresses [201,207,208]. Kim et al. [209] have identified a *Feronia-temperature sensitive* (*fer-ts*) mutant, with G41S substitution in its extracellular domain, that is unable to form root hairs at high temperatures (30 °C). FER controls RHO GTPase (RAC/ROP)-mediated NADPH oxidase-dependent ROS production in root hairs [210]. Kim et al. [209] have observed a reduction in ROS levels in the *fer-ts* mutant at elevated temperatures. Yin et al. [211] have examined the role of *FER2* and -*3* in heat stress tolerance. Interestingly, *FER2* and -*3 m*RNA levels were enhanced under heat stress, and this was dependent on Brassinazole resistant 1 (BZR1), a transcription factor that regulates brassinosteroid (BR) signaling. In addition, chromatin immunoprecipitation real-time quantitative PCR has revealed that BZR1 was bound to the *FER2* and *FER3* promoters and is responsible for regulating their expression. The *fer2/3* mutants showed a decrease in BR-dependent apoplastic H_2_O_2_ accumulation, ROS-detoxification ability, and heat stress tolerance. These results suggest that FER interferes with BZR1-dependent BR signaling and thus ROS production [212].

Additionally, the role of cell wall sensors, FER/HERK1/THE1-4, was also investigated in salt stress responses. Plants impaired in a functional HERK1 and THE1 were severely sensitive to salt stress, comparable to the *fer-4* loss of function mutants. *fer-4* and *herk1the1-4* mutants showed a higher activation of MAP protein kinase 6 and elevated transcript levels of salt-induced marker genes. Moreover, FER alone or with HERK1/THE1-4 demonstrates salt stress-induced pectin de-methyl esterification leading to the activation of downstream signaling events [213]. In conclusion, the few well-investigated CrRLK1Ls are clearly involved in abiotic stress responses.

### 2.9. Leucine-Rich Repeat Extensins (LRXs)

The plant cell wall is composed of a complex network of polysaccharides such as cellulose, hemicellulose, pectin, and also proteins that constitute 20% of the primary cell wall [214,215]. Cell wall proteins (CWPs) are vital for wall modifications, signaling and harbor highly repetitive sequence motifs [216,217,218]. CWPs are categorized into three main classes: proline-rich-proteins (PRPs), glycine-rich-proteins (GRPs), and hydroxyproline-rich-glycoproteins (HRGPs) [216,219,220]. Extensins (EXTs) are cell wall structural proteins that belong to the HRGPs family. They contain a repeating serine-(hydroxyproline)_4_ motif where the serine and hydroxyproline residues undergo glycosylation with often four to five oligoarabinosides becoming attached to hydroxyproline residues and galactose monosaccharides to serine by several glycosyltranferases [221,222,223]. Extensins are basic in nature, with isoelectric points of ∼10, due to their high lysine content, and they form polyproline II helices [216].

LRXs are chimeric, cell wall localized proteins with a conserved N-terminal LRR domain and a highly variable C-terminal extensin domain [224]. LRR domain plays a role in protein–protein interactions while the highly glycosylated extensin domain is probably important for anchoring to polysaccharides such as pectins, in the apoplast [225,226,227]. Arabidopsis contains 11 LRX members that can be subdivided into 2 groups on the basis of their tissue-specific expression. LRX1–LRX7 are expressed in vegetative tissue while LRX8–LRX11 are specifically expressed in pollen. LRX1 and LRX2 are involved in cell wall development and defects in root hairs have been observed for *atlrx1* and *atlrx1/atlrx2* double mutants [228,229]. LRXs bind peptide hormones known as rapid alkalinization factors (RALFs; [230,231,232,233]). The *lrx345* triple mutant, the *fer-4* mutant, and RALF22 or RALF23 overexpressing lines exhibited similar phenotypic aspects such as reduced growth, and they were extremely sensitive to salt stress, indicating that LRX3/4/5 and FER potentially work together in the same pathway. In addition, biochemical data show that LRX3/4/5 is associated with several RALF peptides, including RALF22 and RALF23. This restricts binding of these peptides with FER and its internalization. These findings indicate that LRXs, RALFs, and FER form a signaling hub to coordinate cell wall integrity, plant growth, and salt stress responses [231].

### 2.10. Thaumatin Domain-Containing Receptor-like Kinases

Pathogenesis-related (PR) proteins are produced as a result of the defense response in the host plant upon pathogen invasion [234,235]. These are classified into 17 families (PR1–PR17) based on their mode of action and sequence similarity. Thaumatin-like proteins (TLPs) are structurally similar to thaumatin, a sweet-tasting protein from a rainforest shrub, *Thaumatococcus daniellii* [236]. These proteins belong to the PR5 family and harbor the conserved signature motif G-X-[GF]-X-C-X-T-[GA]-D-C-X-(1,2)-G-X-(2,3)-C [237,238]. Based on their molecular weight, TLPs can be either long (L-type; 22 to 26 kDa) or small (S-type; 18 kDa or less). Their salient characteristic is the presence of 10 and 16 conserved cysteine residues in S-type and L-type TLPs, respectively [239]. These cysteine residues form disulfide bridges which are important for their stability under extreme conditions such as heat and extreme pHs [240]. TLPs can regulate plant responses to various biotic and abiotic stresses, such as pathogenic elicitors, osmotic stress, cold stress, wounding, and plant hormones [241].

Thaumatin-like protein kinases (TLPKs) have a typical RLK structure, with an extracellular thaumatin-like domain that has an antifungal and chitinase activity [52]. The pathogenesis-related 5 RLK (PR5K) from *A. thaliana* was the first characterized TLPK that is majorly expressed in inflorescence stems and roots. It is speculated that it might be a receptor for pathogen-derived elicitors [242]. Three *PR5K* genes are present in Arabidopsis while one gene exists in rice [243]. Their expression can be triggered by various hormones and pathogens [235,244]. Recently, the involvement of AtPR5Ks in ABA-mediated drought stress responses was delineated. Among the three *atpr5k1-1*, *atpr5k2-1*, and atpr*5k3-*1 mutants, a drought tolerance phenotype was detected only for *atpr5k2-1.* On the contrary, *AtPR5K2*-overexpressing plants were found to be hypersensitive to drought stress. Furthermore, AtPR5K2 interacts with the core components involved in ABA signaling, type 2C protein phosphatases ABA-insensitive 1 (ABI1) and ABI2 and the SNF1-related protein kinase 2 (SnRK2.6) proteins [245]. Because this association is abolished in the presence of exogenously applied ABA, AtPR5K2 may function as a negative regulator of ABA signaling under drought conditions [246].

### 2.11. Chitinase (Glycoside Hydrolase)-Type Domain Containing Receptor-like Kinases

Chitinase-related RLK1 (CHRK1) was first isolated from tobacco. The kinase contained an extracellular domain that is closely related to the class V chitinase of tobacco and to bacterial chitinases, though it does not have chitinase activity itself due to the lack of a glutamic acid residue. It is proposed that CHRK1 might take part in defense against pathogens as its activity was induced upon invasion by fungal pathogen and tobacco mosaic virus [247]. By generating a GFP construct and transfecting it into animal cells, the fusion protein resides in the plasma membrane. There are more than 600 RLKs in the Arabidopsis genome, but none of them have been seen to have a chitinase-like sequence [52]. Lee et al. [248] showed that CHRK1 functions in plant development and also regulates cytokinin homeostasis in tobacco. In comparison with other RLK classes, these kinases have received less research attention, and not much is known about their functions in biotic and abiotic stress responses. 

### 2.12. Leaf Rust Kinase 10-like (LRK 10-like)

A novel extracellular recognition domain is present in the LRK10 protein. The *LRK10* gene was initially discovered in wheat (*TaLRK10*) using a homology-based approach based on disease resistance against the leaf-rust-causing fungal pathogen *Puccinia recondite* [249]. The most closely associated Arabidopsis homolog of TaLRK10, *A. thaliana* leaf rust 10 disease-resistance locus receptor-like protein kinase 1 (AtLRK10L1), uses distinct promoters to generate two transcripts, *AtLRK10L1.1* and *AtLRK10L1.2*. Microarray data have disclosed the contribution of *LRK10Ls* in plant growth and stress responses, specifically *AtLRK10L1.2,* in controlling the time of flowering and defense responses against pathogens [250]. Moreover, *LRK10L1.2* participates in ABA signaling at the seedling stage and its localization at the plasma membrane is required for this process as transgenic plants expressing its splicing variant generated a protein that relocated to the endoplasmic reticulum and were seen to be insensitive to high levels of ABA. In addition, *LRK10L1.2* is involved in drought tolerance by facilitating stomatal closure, which was revealed by higher water loss in *lrk10l1-2* mutants under drought conditions. Physiological and molecular functions of the LRK10-like N-terminal domains in abiotic and biotic stress responses have not yet been explored in detail [251].

**Table 1:** Functions of RLKs in different abiotic stress responses.

Genes	Species	Type of RLK	Function	References
Salt Stress				
OsSRK1	*Oryza sativa*	S-receptor protein kinases	Controls leaf development and provides adaptation against salinity	[252]
PsLecRLK	*Pisum sativum*	Lectin	Mitigates salt stress by lowering oxidative damage and increasing the expression of stress-responsive genes thus, retaining ion homeostasis	[253]
SIT1	*Oryza sativa*	Lectin	Negatively regulates salt stress by inducing ethylene and ROS that suppresses plant growth and causes plant death	[254]
MIK2	*A. thaliana*	LRR	Controls the direction of root growth, alters the cell wall structure in the root tip and provides adaptation to salt stress	[255]
OsRLCK253	*Oryza sativa*	RLCK	Interacts with OsSAP11 and prevents yield losses during salt and drought	[256]
GhSIF1	*Gossypium hirsutum*	LRR-RLK	Negative regulator of salt stress responses	[257]
FERONIA	*A. thaliana*	CrRLK1L	Required for restoration of root growth, cell wall stiffness after salt exposure	[208]
LRX 3/4/5	*A. thaliana*	LRX	Forms a signaling network with RALF 22/23 and FER which is pivotal for plant development and adaptation to salt stress	[231]
TaSR	*Triticum aestivum*	LRR–RLK	Participates in salt tolerance by increasing Na^+^ efflux	[258]
PnRLK-1	*Pohlia nutans*	RLCK	Regulates plant sensitivity to ABA and adaptation to salt and oxidative stress	[259]
GsSRK	*Glycine soja*	G-type lectin	Vital for plant response to salt stress	[260]
RLK 7	*A. thaliana*	LRR-RLK	Associates with PAMP-INDUCED SECRETED PEPTIDE 3, activates MPK3/6 and ultimately increases salt stress resistance through maintenance of ionic homeostasis	[261]
STRK1	*Oryza sativa*	RLCK	Confers tolerance against salt stress by activating and phosphorylating Catalase C that maintains H_2_O_2_ balance. Boosts grain yield under salt stress	[262]
PaLectinL16	*Prunus avium*	Lectin	Provides protection against salt stress by increasing the activities of antioxidant enzymes	[263]
RPK1	*A. thaliana* and *Oryza sativa*	Leucine-rich repeat RLK	Negatively regulates salt stress responses, reduces proline synthesis, and inhibits the expression of *SALT OVERLY SENSITIVE 3*	[264]
AtLPK1	*A. thaliana*	Lectin	Functions in salt stress responses by increasing seed germination and cotyledon greening, also participates in pathogen resistance, thus acting as a mediator between abiotic and biotic stress responses	[29]
OsRLCK 311	*Oryza sativa*	RLCK	Regulates stomatal responses under salt stress and binds to aquaporin protein, PIP2;1	[265]
Drought Stress				
HSL3	*A. thaliana*	LRR-RLK	Negatively impacts plant response to moisture deficit conditions through ABA-mediated stomatal closure induced by the generation of H_2_O_2_ in the guard cells	[266]
GUDK	*Oryza sativa*	RLCK	Provides protection against drought stress by activating APETALA2/ETHYLENE RESPONSE FACTOR OsAP37 which triggers the transcription of stress-regulated genes resulting in high yield	[267]
GbRLK	*Gossypium barbadense*	Probable G-type lectin	Crucial for drought and salinity stress tolerance and activation of ABA-dependent signaling events	[268]
CARK6	*A. thaliana*	RLCK	Participates in ABA-mediated drought tolerance	[269]
FON1	*Oryza sativa*	LRR-RLK	Involved in drought stress tolerance in rice by regulating the expression of ABA-responsive genes	[270]
LP2	*Oryza sativa*	LRR-RLK	Acts as a negative regulator in drought response. Interacts with drought-responsive aquaporins and is transcriptionally regulated by C2H2 zinc finger transcriptional factor DROUGHT AND SALT TOLERANCE	[271]
AtLRK10L1.2	*A. thaliana*	LRK 10-like	Takes part in ABA signaling and provides tolerance against drought stress by enhancing stomatal closure	[251]
OsSIK2	*Oryza sativa*	S-RLKs	Reduces the accumulation of H_2_O_2_ under salt stress, participates in dark-induced leaf senescence and plays a vital role under drought conditions	[272]
CRK45	*A. thaliana*	Cysteine-rich RLK	Imparts tolerance against drought stress and controls expression of ABA responsive genes	[273]
AtPR5K2	*A. thaliana*	Thaumatin-like RLK	Plays a negative role in ABA signaling during drought stress by phosphorylating ABI1 and ABI2	[246]
LRK2	*Oryza sativa*	LRR-RLK	Positive regulator of the drought stress response and tiller size in rice	[274]
OsESG1	*Oryza sativa*	S-domain RLK	Participates in drought tolerance by enhancing the activities of antioxidants and expression of stress-regulated genes	[275]
OsSIK1	*Oryza sativa*	LRR-RLK	Inhibits stomatal development in rice leaves which reduces water loss and thereby, providing tolerance against drought stress. Confers adaptation to salt stress by activation of antioxidant enzymes	[69]
ScRIPK	*Saccharum* spp. Hybrids	RLCK	Positively regulates drought tolerance and is a negative regulator of plant defense	[276]
OsRLCK241	*Oryza sativa*	RLCK	Confers tolerance against drought and salt stress by enhancing ROS detoxification, osmolyte production and upregulating the expression of stress-responsive genes	[277]
Oxidative Stress				
ORPK1/ZAR1	*A. thaliana*	LRR-RLK	Positively controls oxidative stress responses and promotes lateral root formation	[278]
XCRLK	*Oryza sativa*	RLCK	Fine tunes ROS levels by detoxifying H_2_O_2_, thus protecting rice plants against oxidative stress	[279]
CRK7	*A. thaliana*	Cysteine-rich RLK	Important for the coordinated response to extracellular but not chloroplastic ROS	[132]
Heavy metal stress				
WAK1	*A. thaliana*	WAK	Involved in tolerance against aluminum toxicity	[111]
WAKL4	*A. thaliana*	WAKL	Plays a vital role in root mineral nutrient responses such as Na^+^, K^+^, Cu^2+^, and Zn^2+^	[280]
OsWAK124	*Oryza sativa*	WAK-RLP	Functions in environmental (heavy) metal stress responses such as Cd^2+^, Cu^2+^, and Al^3+^	[281]
OsWAK11	*Oryza sativa*	WAK	Regulates plant response to metal stress and wounding	[282]
Cold Stress				
GsLRPK	*Glycine soja*	LRR-RLK	Functions in cold tolerance by inducing the expression of cold-inducible marker genes	[70]
CTLK1	*Medicago truncatula*	LRR-RLK	Improves cold tolerance by modulating the expression of antioxidant genes, enzyme activities and proline accumulation	[283]
NDW	*Solanum lycopersicum*	Unknown	Participates in plant growth regulation, cold adaptation and disease resistance against *Botrytis cinerea*	[284]
CTB4a	*Oryza sativa*	LRR-RLK	Confers cold tolerance at the booting stage and improves seed set by regulating pollen fertility and interacts with a beta subunit of ATP synthase, AtpB	[285]
OsRLCK48	*Oryza sativa*	RLCK	Its expression is downregulated under cold stress	[30]
Heat stress				
TMS10	*Oryza sativa*	LRR-RLK	Plays a role in tapetal degeneration and male fertility under high temperatures	[286]
ERECTA	*A. thaliana*	LRR-RLK	Introduction of *ERECTA* gene in *Pinellia ernate* disrupted the summer dormancy. It is crucial for preventing plant cells from cellular damage caused by high heat and positively regulates transpiration efficiency in rice and tomato	[287,288]
AtPXL1	*A. thaliana*	LRR-RLK	Interacts with histidine-rich dehydrin1, light-harvesting protein complex I and is involved in signaling under cold and heat stress	[289]
FER	*A. thaliana*	CrRLK1L	Required for root hair development under elevated temperatures	[209]
CaHSL1	*Capsicum annuum*	LRR-RLK	Provides thermotolerance against high temperature and high humidity	[290]
CaWAKL20	*Capsicum annuum*	WAKL	Negative regulator of plant thermotolerance as it suppresses the expression of ABA-responsive genes	[291]
TaXa21	*Triticum aestivum*	LRR-RLK	Positively mediates high temperature plant resistance to *P. striiformis* f. sp. *Tritici* by interacting with TaWRKY76 and TaWRKY62	[292]

## 3. Biological Functions of RLKs in Abiotic Stress Responses

Direct sensing of abiotic stress by RLKs may occur via their extracellular domains, which transduce the information to the cytoplasmic compartment to initiate appropriate downstream responses [50]. Proper signaling of the RLKs requires an intact plasma membrane around the stress-exposed cell. In order to identify candidate RLKs involved in stress sensing, (a) the expression levels of an *RLK* gene might respond to a stress treatment a, (b) manipulation of the *RLK* mRNA or RLK protein level in a plant could alter stress-induced responses which occur in the un-manipulated plants, and (c) manipulation of the RLK levels alters the stress resistance phenotype of the plant in comparison with unmanipulated plants. In all three cases, the correlation between input and output can be positive or negative, depending on whether the RLK is an activator or repressor of the stress response. Basically, these three criteria were used to identify RLKs involved in abiotic stress resistance. We describe the well-investigated RLK from different plant species that are involved in salt, drought, oxidative, temperature and metal stress.

### 3.1. Salt Stress

**Figure 2:**
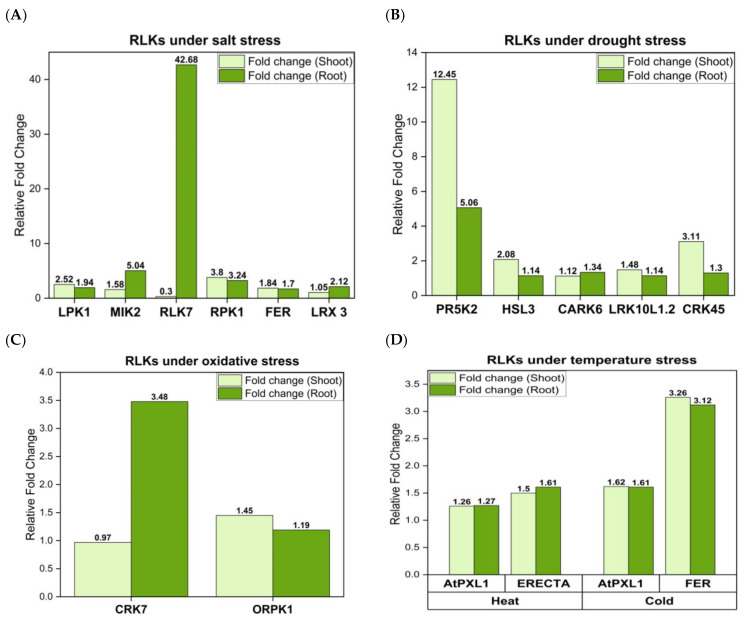
Fold change of *RLK* mRNA levels in shoots and roots under different stress treatments in Arabidopsis. (**A**) Salt stress, (**B**) drought stress, (**C**) oxidative stress, and (**D**) temperature stress (heat and cold). The above graphs represent maximum relative fold change values at a particular time point after stress exposure. Expression of these RLKs under different stress conditions was analyzed by Arabidopsis eFP Browser at BAR website (http://bar.utoronto.ca/efp/cgi-bin/efpWeb.cgi, accessed on 18 August 2023).

Salt stress is a major environmental constraint that affects 20% of irrigated land leading to germination failure, reduced growth, and yield losses [1,293,294]. Excess salt causes ionic (mainly due to Na^+^, Cl^−^, and SO_4_^2−^) and osmotic stresses, as well as secondary stresses such as oxidative stress and nutritional deficiencies [295]. Plants are classified as halophytes or glycophytes depending upon their ability to tolerate salinity. Halophytes have the capacity to tolerate high salt concentrations (400 mM NaCl), while glycophytes are sensitive to high salt conditions [296]. In order to maintain their ion homeostasis, plants must sense excess salt in the apoplast (cf. Table 1). OsSRK1 is an atypical S-receptor-like kinase whose expression is enhanced upon ABA, salt, and polyethylene glycol (PEG) 4000 treatment. *OsSRK1*-overexpression (*OsSRK1*-OX) plants have been shown to be sensitive to ABA but, interestingly, were found to be highly tolerant to salt stress in comparison with the wild type. The mechanism underlying OsSRK1-mediated salt tolerance is unclear but can be attributed to the induction of a set of genes involved in dehydration, such as *DEHYDRATION RESPONSIVE ELEMENT-BINDING*, *OsDREB1A*. This makes SRK1 an interesting candidate in rice agriculture [252]. LecRLKs have been extensively studied for their role in environmental stress conditions. Vaid et al. [253] have investigated the role of *PsLecRLK*, an L-type LecRLK from *Pisum sativum*, under salinity stress conditions. When the expression pattern of *PsLecRLK* was analyzed in response to salinity, temperature stress, wounding and hormone applications, an 80-fold induction was observed under salt stress. Overexpression of *PsLecRLK* in transgenic tobacco plants resulted in a salt-tolerant phenotype which was evident from higher biomass, germination rate, growth, and photosynthetic pigment content. Moreover, estimation of malondialdehyde, 3,3′-diaminobenzidine and nitroblue tetrazolium staining indicated lesser ROS levels and lower membrane damage in these plants. *PsLecRLK* overexpression resulted in a lesser accumulation of Na^+^ ions and a higher expression of water channels and transporters involved in ion homeostasis under salt stress. This indicates that *PsLecRLK* stabilizes the membranes and ion homeostasis under salt stress by stimulating transport processes across the plasma membrane. In another study, *Salt Intolerance 1* (*SIT1*) from rice was shown to negatively regulate salt tolerance. This is expressed in root epidermal cells and becomes activated under salt stress. SIT1 activates MAPK3 and MAPK6 leading to ethylene generation. Finally, SIT1 enhances the ROS generation that inhibits growth and causes plant death under salinity, which in turn is dependent on MAPK3/6 and ethylene signaling in *A. thaliana* [254]. Furthermore, maintenance of cell wall integrity is crucial for plants in order to acclimatize to saline conditions [297]. MDIS1-INTERACTING RECEPTOR LIKE KINASE2 (MIK2) of the sub-family XIIb of LRR-RLKs is vital for regulating the responses activated by the suppression of cellulose biosynthesis. In addition, MIK2’s role in salt stress tolerance is dependent on THE1, though it does not require THE1 in order to provide resistance to *Fusarium oxysporum* in roots (Figure 2) [255]. Apparently, this is an interesting starting point from which to experimentally dissect the signaling leading to abiotic and biotic stress responses. 20⁄AN1 zinc-finger domain-containing stress-associated proteins (SAPs) are central for imparting tolerance against abiotic stresses [298]. Giri et al. [256] have shown that *OsSAP11* interacts with *OsRLCK253* at the nuclear membrane and only weakly at the plasma membrane. By generating overexpressor lines, it was observed that *OsSAP11* and *OsRLCK253* enhanced plant survival and reduced yield losses under salt and drought stress. Transcript profiles indicated the upregulation of genes involved in the biosynthesis of antioxidant compounds such as anthocyanin and carotenoids, which might be a reason for the protection of these plants against different stresses. Stress-induced factor genes (SIF1–SIF4) belong to a multifunctional kinase family which is involved in biotic and abiotic stress responses in Arabidopsis. Phylogenetic analysis revealed six LRR-RLKs in cotton that are homologous to Arabidopsis SIFs and, among these, GhSIF1 showed a 46–47% amino acid sequence similarity. Yuan et al. [257] have characterized the role of *GhSIF1* in salt tolerance by knocking it out using a virus-induced gene silencing system in cotton. The plants were better protected against salt stress in the transient assay system and they exhibited enhanced growth, lower electrolyte leakage, and higher chlorophyll content than the control plants. These results demonstrate that *GhSIF1* negatively regulates salt tolerance. Apparently, the participation of RLKs in salt stress tolerance ranges from the activation of general responses to abiotic stress to more specific responses stabilizing the ion homeostasis. This includes alterations in gene expression and enzyme activity.

K^+^, the most prevalent cation in plants, is crucial for the maintenance of cell turgor, carbohydrate metabolism, starch synthesis, osmoregulation, and enzyme activation [299,300]. Moreover, K^+^ uptake and transport play a vital role in providing tolerance against various abiotic stresses, such as drought and salinity. K^+^ enhances the activity of antioxidant enzymes, thereby decreasing the accumulation of ROS and also regulates stomatal opening under water deficient conditions [301]. Salinity tolerance is acquired by establishing K^+^ homeostasis and a low Na^+^/K^+^ ratio in the plant [302,303]. When plants are exposed to salt stress, Na^+^ competes with K^+^ for uptake sites at the plasma membrane [304]. As a result, membrane integrity is disrupted due to depolarization that leads to the efflux of K^+^ via K^+^ channels (depolarization-activated outward-rectifying K^+^ channels) [305].

Salt stress causes an increase in cytosolic free calcium (Ca^2+^) concentration [306] which is sensed by a salt overlay sensitive (SOS) pathway that consists of SOS3, SOS2, and SOS1 [307]. SOS3, a myristoylated Ca^2+^ binding protein senses the elevation in Ca^2+^ levels and binds with Ca^2+^ which activates SOS2 or calcineurin-B-like protein (CBL)-interacting protein kinase (CIPK24), a serine/threonine protein kinase. The SOS3–SOS2 complex activates SOS1, a Na^+^/H^+^ antiporter, which is responsible for the extrusion of Na^+^ from the cytosol [295,308]. CBLs interact with plant Ser/Thr kinases, namely the CIPKs, which are homologous to yeast and animal sucrose non-fermenting (SNF) protein kinases [309]. The CBL–CIPK network has been extensively studied under K^+^-limiting conditions in Arabidopsis. Exposure of plants to long term salt stress decreases K^+^ content and stimulates K^+^ transporters. CBL1/CBL9 associates with CIPK23 and targets it to the plasma membrane of root cells to phosphorylate a voltage-gated high-affinity K^+^ channel, Arabidopsis K^+^ transporter 1 (AKT1). Its activation enhances K^+^ uptake [310].

### 3.2. Drought Stress

**Figure 3:**
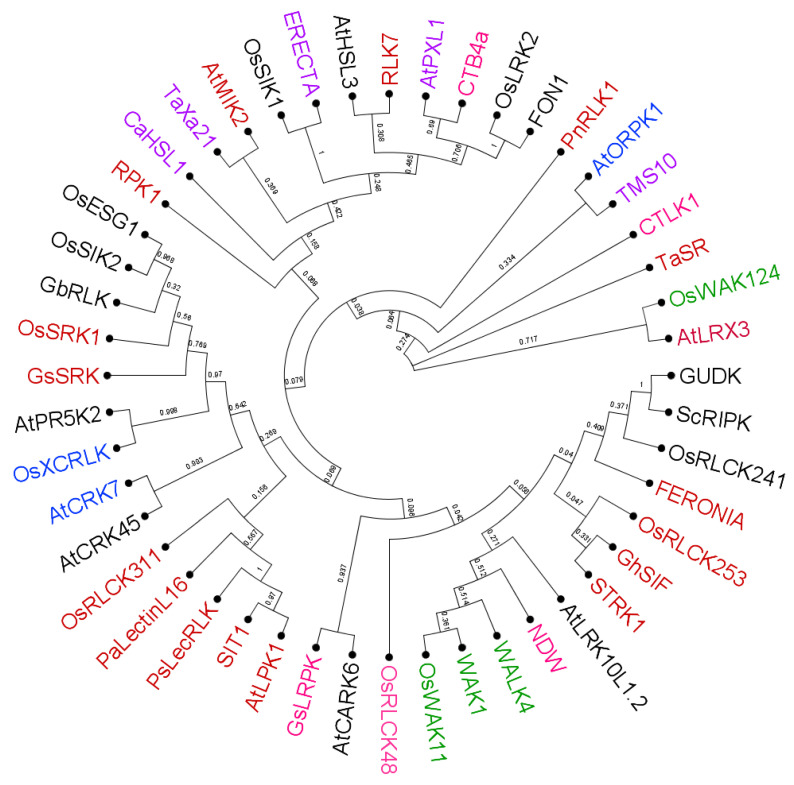
Phylogenetic tree for the RLKs involved in various abiotic stresses in different plant species. Different colors represent various stress conditions in which the RLKs mentioned in Table 1 are involved. The RLKs colored in red are involved in salt stress; green in (heavy) metal stress; blue in oxidative stress; violet in heat stress; pink in cold stress; and black in drought stress. Multiple sequence alignment of their amino acid sequences was performed by MUSCLE program. Phylogenetic tree was constructed by the neighbor-joining method, with 1000 bootstrap replications using the MEGA 11.0.13 software (https://www.megasoftware.net, accessed on 21 August 2023/; Kumar et al. [330]).

Drought is an inevitable environmental factor that impacts 64% of land area worldwide and impedes photosynthesis, stomatal movement, ion uptake, metabolism, and can even cause plant death [311]. It is well documented that plants have developed phenotypic plasticity through intricate signaling networks involving RLKs [312]. Several RLKs have been linked to drought stress responses [52] (Figure 3). ABA is an essential stress hormone that can regulate the expression of osmotic stress-responsive genes and facilitates stomatal closure under drought stress [313]. HAESA-LIKE3 (HSL3) is an LRR-RLK that is induced by ABA, H_2_O_2_ and water deficiency. In the *hsl3* mutant, over-accumulation of H_2_O_2_ and a higher net flow of anions in the guard cells was observed, resulting in the closure of stomata and thereby providing tolerance against drought stress. HSL3 acts as a negative regulator of drought stress by tweaking the levels of H_2_O_2_ in the guard cells [266]. ERECTA (ER), another LRR-RLK from Arabidopsis has been implicated in development and disease protection. However, its role in water deficiency has not been explored. Recently, two ER genes from *Sorghum bicolor* L., *SbER1–1* and *SbER2–1* were cloned, and their expression was evaluated under moderate and severe drought stress. *SbER2* transcript levels were greatly enhanced in response to drought, and a *35S::SbER2–1-eGFP* fusion protein was localized on the plasma membrane and in chloroplasts. This suggests a role for SbER2-1 in photosynthesis. Overexpression of *SbER2–1* in Arabidopsis and maize resulted in higher drought tolerance of the shoot. Differentially expressed gene analysis of maize lines overexpressing *SbER2–1* depicted genes enriched in glutathione metabolism in leaves and phenylpropanoid biosynthesis in stems. Moreover, these plants also showed higher lignin content as well as water use efficiency, indicating that *SbER2–1* is a vital target for genetic engineering in order to produce plants that are resistant to drought stress [314]. An RLCK, GROWTH UNDER DROUGHT KINASE (GUDK) is responsible for imparting drought tolerance under both vegetative and reproductive stages in rice. This was demonstrated by the use of *gudk* loss-of-function mutant lines as they are sensitive to salinity, osmotic stress, and ABA at the seedling stage. The grain yield was reduced in these lines under well-watered conditions and drought stress. Furthermore, phosphoproteomics unraveled the transcription factor APETALA2/ETHYLENE RESPONSE FACTOR, OsAP37 as a target of GUDK which, in turn, activates genes involved in photosynthesis, carbon metabolism, and drought tolerance [267]. Another study by Zhao et al. [268] provides insights into the mechanism by which an RLK gene from cotton, *GbRLK*, imparts tolerance against various abiotic stresses including drought. The *GbRLK* promoter was fused to the *β-glucuronidase* (*GUS*) gene and histochemical staining indicated induction of GUS levels upon exposure to ABA, PEG, salt, and *Verticillium dahlia* infection. Overexpression of *GbRLK* in Arabidopsis reduced water loss and these transgenic plants are more tolerant towards drought. Furthermore, these lines showed hypersensitivity to ABA, indicating that GbRLK is involved in ABA-mediated signal transduction. Additionally, the upregulation of various stress-responsive genes, *RESPONSIVE TO DESICCATION* (*AtRD20*, *AtRD22*, and *AtRD26*) and antioxidant genes such as *CATALASE 1* (*AtCAT1*), *COPPER CHAPERONE FOR SUPEROXIDE DISMUTASE* (*AtCCS*), and *COPPER/ZINC SUPEROXIDE DISMUTASE 2* (*AtCSD2*) was also detected, which can mitigate the adverse effect of these stresses by detoxification of ROS. Another RLCK VIII subfamily member in Arabidopsis, cytosolic ABA receptor kinase 6 (CARK6), participates in the regulation of germination and root growth and stimulates drought resistance by interacting with ABA receptors. Thus, further studies are needed to unravel the molecular mechanism underlying its role in ABA signaling and to manipulate it for crop improvement [269]. These examples demonstrate that those RLKs which are involved in drought tolerance, either crosstalk with ABA signaling or—in the case of CARK6—interact directly with ABA receptors.

### 3.3. Oxidative Stress

Multiple lines of evidence support the dual role of ROS as both toxic by-products of aerobic metabolism as well as vital signaling molecules for inter- and intracellular communication in plants [315,316]. ROS generation is stimulated by various environmental stresses that include excessive light, wounding, ozone, drought, UV irradiations, pathogen invasion, low and high temperatures, and heavy metals [317]. Oxidative stress occurs as a result of the overproduction and accumulation of ROS in various organelles. It can cause cellular damage to biomolecules such as DNA, proteins, and lipids that may even lead to cell death [318,319]. RLK signaling often results in apoplastic ROS generation, indicating an intricate connection between RLKs and ROS burst [320]. Because ROS production is a general response to many abiotic and biotic stresses, it is not surprising that elevated ROS levels have been described for many of the activated RLKs described here. Recently, the role of a previously uncharacterized LRR-RLK, *Oxidative-stress Related Protein Kinase 1* (*ORPK1*) from Arabidopsis was dissected by Yang and Jiang [278]. *AtORPK1* mutants were shown to be extremely sensitive to oxidative stress while the overexpression of *AtORPK1* enhanced the oxidative stress tolerance of transgenic plants. The expression of antioxidant enzyme genes such as *IRON SUPEROXIDE DISMUTASE* (*FeSOD*), *CATALASE1* (*CAT1*), and *ASCORBATE PEROXIDASE1* (*APX1*) was downregulated in *orpk1* mutants and upregulated in overexpression lines. Examining how this regulation occurs at the molecular level is an interesting task for future studies. Fluorescent resonance energy transfer (FRET) analyses have demonstrated that AtORPK1 dimerizes at the plasma membrane upon ligand binding. This results in the autophosphorylation of the complex and endocytosis. In the endosome/prevascular compartment, the C-terminal kinase domain of AtORPK1 interacts directly with AtKAPP. Trafficking and binding to AtKAPP is essential for the activation of downstream antioxidant genes [278]. Taken together, these results suggest that AtORPK1 positively regulates oxidative stress signaling in plants. Analysis of rice proteomes revealed a set of differentially expressed proteins upon infection with *Xanthomonas oryzae pv. Oryzicola* (*Xoc*), with the *Xoc*-associated receptor-like kinase (XCRLK) a candidate among these, one whose role in biotic and abiotic stress responses has been investigated. mRNA levels of *XCRLK* were seen to be significantly upregulated by ABA, indole acetic acid, and H_2_O_2_ application, implying that it might participate in the response to several stresses and phytohormones. Overexpression of *XCRLK* has resulted in enhanced resistance towards *Xoc* and these plants accumulated lesser H_2_O_2_ compared with wild-type seedlings. Additionally, the expression of the resistance genes, *PHENYLALANINE AMMONIA-LYASE 1* (*PAL1*) and *PR5,* and the oxidation-related genes, *WRKY77* and *WRKY13,* was seen to be higher in *XCRLK*-overexpressing transgenic plants, indicating that XCRLK is essential for providing resistance against *Xoc* and enhancing the antioxidant ability in rice [279]. In summary, the broad spectrum of stress responses with ROS participation makes it difficult to define a specific role of RLKs in abiotic tolerance responses. In many cases, ROS production is far downstream of stress perception. However, AtORPK1 provides an example for a mechanism of how an RLK directly controls oxidative stress resistance.

### 3.4. Temperature Stress

The earliest physiological change that occurs in response to low temperature is the reduction in membrane fluidity that results from alterations in lipid composition [321]. Moreover, it can also lead to the rearrangement of the cytoskeleton, dehydration of cells and tissues, electrolyte leakage, Ca^2+^ influx, ultrastructural modifications in photosynthetic apparatus, and electron transport [322]. In recent years, studies on the identification of plant sensors and transcriptional networks involved in low-temperature sensing have been gaining momentum, as this knowledge will aid in the development of crops with improved winter resilience [323]. An increasing number of studies have suggested a role for RLKs in the regulation of cold adaptation. CBFs or DREBs regulate the expression of *COR* genes and play a pivotal role in cold acclimation [324]. Transcription levels of Cold tolerance LRR-RLK1 (*CTLK1*) in *Medicago truncatula* and *M. falcate* were upregulated by cold treatment and the loss of *MtCTLK1* resulted in lower survival rates, while its overexpression enhanced the survival rate and lowered the 50% ion leakage after freezing stress. Furthermore, *MtCTLK1* overexpressors had higher antioxidant enzyme activities and proline levels along with higher mRNA levels of associated genes. Additionally, the induction of CBF*s* and CBF-dependent cold-responsive genes was lower in the *mtctlk1* mutants but higher in the *MtCTLK1*-overexpressor plants, indicating that *MtCTLK1* protects *M. truncatula* from cold stress. The above evidence suggests a positive role for *MtCTLK1* in providing tolerance against cold stress through regulation of the CBF pathway, antioxidant defense system, and proline accumulation [283]. Using forward genetic screens, an LRR-RLK, *CTB4a* (*cold tolerance at booting stage*), was cloned and was found to play a role in seed setting by increasing the pollen fertility during cold stress in rice. Biochemical assays revealed the interaction between CTB4a and AtpB, the beta subunit of ATP synthases, demonstrating that this association is crucial for the synthesis of ATP. The ATP might provide energy for improving seed setting, and thus yield, under cold stress conditions [285].

The involvement of RLKs in plant thermotolerance and their underlying mechanisms is poorly documented, although a few studies have elucidated how RLKs buffer the detrimental effects of high temperatures. The LRR-RLKs *Thermo-Sensitive Genic Male Sterile 10* (*TMS10*) and its close homolog *TMS10-Like* (*TMS10L*), have redundant functions in regulating male fertility under variable temperatures. *tms10* mutant plants did not produce viable pollen grains and showed male sterility under high temperatures (>24 °C) because of abnormalities in tapetal degeneration and pollen wall formation. However, it is partially fertile under low temperatures (23–24 °C). Interestingly, *tms10 tms10l-1* and *tms10 tms10l-2* double mutants displayed male sterility under both low and high temperatures. These observations suggest that TMS10L and TMS10 function together to safeguard the effect of changing temperatures on male fertility in rice [286]. Previous studies have documented the importance of multi-functional *ER* genes in plant development and in response to environmental changes [325]. To break summer dormancy and to provide heat tolerance, Juneidi et al. [287] introduced a heat responsive *ER* gene into the Chinese medicinal plant, *Pinellia ternata*. The transgenic lines were better adapted to heat stress. Moreover, higher leaf stomatal conductance, water-use efficiency, and carbon assimilation were detected in *ER* overexpressor genotypes on exposure to heat stress than in the controls. These results were confirmed under field conditions, where the plants were exposed to 35 °C for 90 days, and highlight the thermo-tolerant ability of ER in *P. ternata*. In conclusion, although there is sufficient evidence for the involvement of RLKs in cold and heat stress adaptation, the underlying molecular mechanisms are poorly investigated. The signaling pathway for plant adaptation to cold was quite intensively studied and contains well defined signaling components, while adaptation to heat is more complex and diversified. Therefore, the interference of RLKs that leads to cold adaptation is probably easier to investigate than that which leads to heat adaptation.

### 3.5. Metal Stress

Although the metals and metalloids collectively known as heavy metals (HMs) play significant roles in diverse biological processes, such as metabolic reactions and antioxidant defense systems, extensive exposure to certain metals can be lethal to microorganisms and plants [326]. These are taken up by the plants, which results in the inhibition of germination, root elongation, reduced crop yields and the generation of ROS [327]. Plants have acquired various strategies to tolerate and detoxify metals and heavy metals and these are mediated by a nexus of signaling pathways, including RLKs. *WAK1* has been reported to be a key gene responsible for imparting tolerance against aluminum (Al^3+^) toxicity in Arabidopsis. Al^3+^ exposure results in the upregulation of *WAK1* mRNA and protein levels after 3 and 6 hours, respectively. Moreover, overexpression of *WAK1* leads to a 3-fold increase in root growth as compared with wild-type plants in the presence of Al^3+^ [111]. Analysis of the *WAKL4* promoter fused with the GUS reporter gene indicated that it was strongly activated by various mineral nutrients, including Na^+^, K^+^, copper (Cu^2+^), nickel (Ni^2+^), and zinc (Zn^2+^). A T-DNA introduced into the promoter region of the *WAKL4* gene inhibited its expression by Na^+^, K^+^, Cu^2+^, Zn^2+^ but, interestingly, not by Ni^2+^. *Wakl4–1* mutants are hypersensitive to Na^+^, K^+^, Cu^2+^, and Zn^2+^, as indicated by the reduction in root lengths, while they are tolerant towards toxic levels of Ni^2+^. In addition, *WAKL4* is essential for the expression of Zn^2+^ transporter genes under Zn^2+^ limiting conditions. Overall, these results highlight the importance of *WAKL4* in plant mineral responses [280]. Yin and Hou [281] investigated the expression patterns of *OsWAK124* under numerous stress environments, including NaCl, AlCl_3_, CuSO_4_, and CdSO_4_. GUS staining revealed that, under normal conditions, *OsWAK124* primarily expresses at the shoot–root junction. However, on exposure to the above-mentioned stresses, its promoter activity is also induced in non-meristematic tissues, such as leaf, stem, and root. Furthermore, overexpression of *OsWAK124* rendered rice plants more resistant to Al^3+^, Cu^2+^ and Cd^2+^, indicating that it participates in (heavy) metal stress responses. Additionally, *OsWAK11* contributes to Cu^2+^ detoxification when plants are exposed to excess amounts of Cu^2+^. By knocking out of *OsWAK11*, the plants became hypersensitive to Cu^2+^ due to the higher uptake of Cu^2+^ by *OsWAK11*-RNAi seedlings into the cytoplasm. Intriguingly, there was also a significant decrease in Cu^2+^ levels in the pectin and hemicellulose fractions, suggesting that *OsWAK11* controls the Cu^2+^ binding ability in the cell wall. It has been reported that the degree of pectin methyl esterification (PME) is negatively related to PME activity in Arabidopsis [328]. *OsWAK11*-RNAi exhibited greater cell wall methyl esterification than wild-type plants, resulting in fewer transcription levels of the PME encoding gene *OsPME14* under normal and Cu^2+^-stressed conditions. Moreover, disruption of *OsPME14* resembled the phenotypes of *OsWAK11*-RNAi lines under Cu^2+^ stress, indicating the possibility of *OsPME14* as a downstream component of *OsWAK11*. In conclusion, under Cu^2+^ toxicity, plants activate *OsWAK11* to modify the properties of the cell wall so that the immobilization of Cu^2+^ in the cell wall is enhanced [329].

## 4. Conclusions and Perspectives

RLKs act as molecular hubs for communication between cells and the extracellular environment, which is essential for the coordinated development and growth of a plant under stress conditions. Great progress has been made in characterizing RLKs but our knowledge about their specificity and downstream components in abiotic stress responses is still fragmentary. Although great attention has been given to studying the role of LRR-RLKs in biotic stress responses, there is a pressing need to integrate these less-studied kinases into abiotic stress research as this will reveal novel stress-responsive components which can be leveraged for engineering crops with broad-spectrum resistance.

The involvement of RLKs in various biotic stress responses is well documented, while much less is known about their involvement in abiotic stress responses. This raises the question as to whether this is due to the lack of experiments or whether RLKs are more involved in biotic stress responses. Therefore, it is important to identify the specific ligands that activate the RLK-induced signaling pathways. Besides protein–protein interactions in or at the plasma membrane after receptor activation, some of the downstream signaling molecules might be involved in multiple biotic and abiotic stress responses. This includes Ca^2+^, ROS and MAPKs, although different stimuli establish, e.g., different Ca^2+^ signatures or produce ROS in different compartments. Therefore, it might be important to understand which of the downstream components are involved in establishing the crosstalk between the signaling pathways without losing the specificity of the response.

The phylogenetic tree (Figure 3) demonstrates that there is little relationship between the evolution of the RLKs and the abiotic stress by which it they can be activated. Closer inspection of the tree might help to identify novel RLKs in different plant species involved in a specific stress response, or vice versa.

Most of the studies have examined a plant’s response to one stress at a time, but under natural conditions, plants face multiple abiotic and biotic stresses simultaneously, thus, it becomes imperative to understand how RLKs integrate signals from diverse stimuli to fine-tune their defense strategies and to allocate resources effectively. Powerful molecular tools, such as single-cell-omics, cellular and subcellular imaging, metabolomics, and phenomics analyses, will be indispensable in order to gain an in-depth understanding of kinase-mediated signal transduction pathways under abiotic stresses [331]. Genetic studies have revealed only a handful of ligand–receptor pairs and most of the RLKs are still orphans. Ligand identification is a challenging task due to high redundancy, but is nonetheless pivotal if we are to unravel their functions and the intricate signaling web they regulate. Although research on model plants, Arabidopsis and rice have contributed significantly to our understanding of the molecular mechanisms underpinning responses to various stressors, identification of RLKs in other crops will help us to design strategies for crop improvement specifically tailored to their genetic backgrounds. Furthermore, dissection of the interaction partners of RLKs when responding to biotic and abiotic stresses through phosphoproteomics may be fruitful for targeted genetic manipulations and the design of crops with enhanced resilience to multiple stresses.

## Data Availability

Not applicable.
